# The application of Aptamer in biomarker discovery

**DOI:** 10.1186/s40364-023-00510-8

**Published:** 2023-07-19

**Authors:** Yongshu Li, Winnie Wailing TAM, Yuanyuan Yu, Zhenjian Zhuo, Zhichao Xue, Chiman Tsang, Xiaoting Qiao, Xiaokang Wang, Weijing Wang, Yongyi Li, Yanyang Tu, Yunhua Gao

**Affiliations:** 1 Center for Advanced Measurement Science, National Institute of Metrology, Beijing, China; 2 Shenzhen Institute for Technology Innovation, National Institute of Metrology, Shenzhen, China; 3 Law Sau Fai Institute for Advancing Translational Medicine in Bone and Joint Diseases (TMBJ), School of Chinese Medicine, Hong Kong Baptist University, Hong Kong SAR, China; 4 State Key Laboratory of Chemical Oncogenomic, Peking University Shenzhen Graduate School, Shenzhen, China; 5 Laboratory Animal Center, School of Chemical Biology and Biotechnology, Peking University Shenzhen Graduate School, Shenzhen, 518055 China; 6 Department of Anatomical and Cellular Pathology, State Key Laboratory of Translational Oncology, The Chinese University of Hong Kong, Hong Kong, China; 7 Department of Pharmacy, Shenzhen Longhua District Central Hospital, Shenzhen, China; 8 Shantou University Medical College, Shantou, China; 9 Research Center, Huizhou Central People’s Hospital, Guangdong Medical University, Huizhou City, China

**Keywords:** Aptamer, Biomarker discovery, SELEX, SOMAScan, Cardiovascular diseases, cancer, Neurodegeneration-related diseases

## Abstract

Biomarkers are detectable molecules that can reflect specific physiological states of cells, organs, and organisms and therefore be regarded as indicators for specific diseases. And the discovery of biomarkers plays an essential role in cancer management from the initial diagnosis to the final treatment regime. Practically, reliable clinical biomarkers are still limited, restricted by the suboptimal methods in biomarker discovery. Nucleic acid aptamers nowadays could be used as a powerful tool in the discovery of protein biomarkers. Nucleic acid aptamers are single-strand oligonucleotides that can specifically bind to various targets with high affinity. As artificial ssDNA or RNA, aptamers possess unique advantages compared to conventional antibodies. They can be flexible in design, low immunogenicity, relative chemical/thermos stability, as well as modifying convenience. Several SELEX (Systematic Evolution of Ligands by Exponential Enrichment) based methods have been generated recently to construct aptamers for discovering new biomarkers in different cell locations. Secretome SELEX-based aptamers selection can facilitate the identification of secreted protein biomarkers. The aptamers developed by cell-SELEX can be used to unveil those biomarkers presented on the cell surface. The aptamers from tissue-SELEX could target intracellular biomarkers. And as a multiplexed protein biomarker detection technology, aptamer-based SOMAScan can analyze thousands of proteins in a single run. In this review, we will introduce the principle and workflow of variations of SELEX-based methods, including secretome SELEX, ADAPT, Cell-SELEX and tissue SELEX. Another powerful proteome analyzing tool, SOMAScan, will also be covered. In the second half of this review, how these methods accelerate biomarker discovery in various diseases, including cardiovascular diseases, cancer and neurodegenerative diseases, will be discussed.

## Introduction

A biomarker is a measurable substance or characteristic in an organism that can indicate the presence or severity of a disease, infection, or other physiological state [1]. Biomarkers can be found in various bodily fluids, including blood, urine, and cerebrospinal fluid, as well as in tissues and organs. The detection of the abnormal level of biomarkers can character the specific physiological and pathological differences in living organisms. Thus, biomarkers are essential for clinical medicine, drug development and bench-to-bedside research. In clinical applications, biomarker measurement can provide information on disease progression for disease diagnosis, patient prognosis, and risk forecasting. For example, the level of natriuretic peptide and cardiac troponin are effective clinical biomarkers for the diagnosis and prognosis of heart failure and acute myocardial infarction [2]. The level of C-reactive protein (CRP) can reflect an inflammation status of the human body and indicate the potential risks of infection, chronic inflammatory disease (such as rheumatoid arthritis or lupus), heart disease, and a second heart attack [3]. Traditional cytotoxic chemotherapies usually target the process of cell division without a specifying targeted molecule. Hence, they are less efficient to cancer cells and have more side effects to normal cells. The biomarker-based drug development strategy focuses on specific proteins promoting cancer cell growth and survival, avoiding off-target effects on normal cells [4]. One of the most prominent examples of targeted therapy is the use of anti-HER2 antibodies to treat HER2-overexpressed breast and gastric cancers. Aberrant activation of human epidermal growth factor receptor 2 (HER2) has been found to promote carcinogenesis in subtypes of breast cancer and gastric cancer. Direct inhibition of HER2 has been considered a cornerstone and adequate therapeutic in HER2-overexpressed cancers with improved potency and fewer side effects [5]. Although great efforts have been made and certain achievements have been realized, they are still unsatisfied in facing the growth in demand. Regarding biomarker-based diagnosis and therapy, the overall progress is still slow due to the lack of effective methods for biomarker discovery [6].

Assessment of differential protein expression has been one of the most common approaches in proteomic biomarker discovery. By comparing the protein expression profile between healthy and diseased samples, protein uniquely expressed or altered expression profiles in disease states may be identified. Proteomics analysis could be further divided into mass spectrometry (MS) based and antibody-based assays [7, 8]. As a powerful proteomic analysis method, MS can provide in-depth analysis of peptide or protein content in complex mixtures like blood sample and facilitate biomarker discovery [9]. But still, there are some limits to the practical application of MS for protein biomarker exploration. Complex sample preparation, such as fractionation by high-performance liquid chromatography (HPLC) and enzyme digestion, is required prior to MS. False positive signals during the sample preparation process [6]. False positive signals may be introduced during the sample preparation process [10, 11]. Unmatched sensitivity levels also hold back the application of MS in biomarker discovery, especially from body fluids. Putative biomarkers are subject to dilution and clearance after entering the circulation system, resulting in low concentrations of analysts ranging from 50pg/ml to 5ng/ml [12, 13]. In contrast, the antibody-based method excels in both sensitivity and specificity, hence is extensively employed for the detection of a specific protein. The antibody-based assay can detect target protein biomarkers in the sub-nM range, such as enzyme-linked immunosorbent assay (ELISA) and enzyme immunoassay, which lead to the most extensive usage in protein detection [14]. However, when it comes to biomarker discovery, the specificity of antibody can be a double-edged sword. The classical double-antibody sandwich method in ELISA has been treated as a standard sensitive technology for protein concentration definition. This mechanism of action is the usage of two different antibodies to capture and recognize the same protein to enhance specificity, which means only a single target protein can be detected at a single time. More and more works support that a single cytokine or growth factor biomarker may hardly reflect the dynamic disease process. Like other planar arrays, an antibody array is developed as a multiplexed platform in high-throughput detection [15]. Although multiplexed platform for high-throughput detection has been developed to accelerate biomarker discovery, currently available antibodies are inadequate for covering full human proteome, hindering novel biomarker discovery. Other antibody inherent drawbacks, such as cross-reactivity, poor reproducibility, complicated synthesis procedures and expensiveness, have restricted their efficiency. Moreover, the discovery of tumor-associated antigens and other disease markers is usually short of appropriate and certain biomarkers because it is commonly recognized as an open-end problem. As more and more researches reveal that a single cytokine or growth factor does not suffice to explain the complete pathogenic mechanism, aptamers have emerged as novel baits for effective and efficient protein detection.

**Fig. 1:**
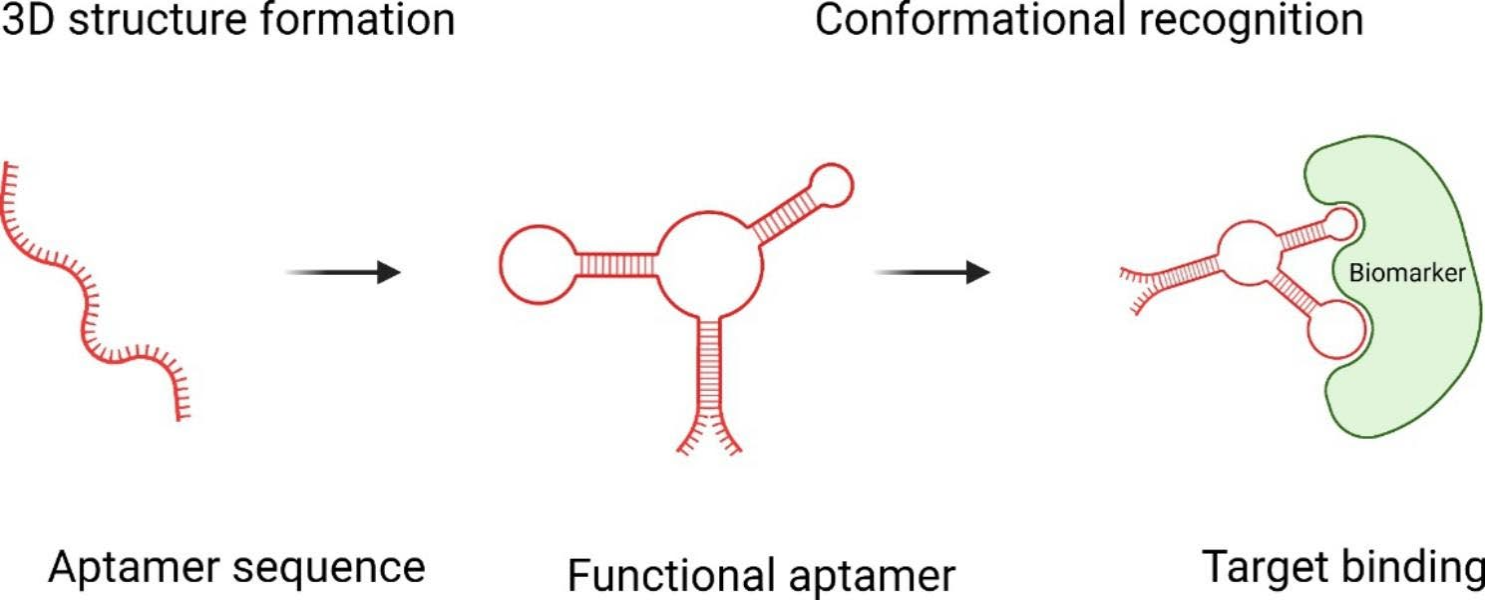
An aptamer binds to the target. Single-stranded aptamer is folded to be functional. It binds to its target whether protein, RNA or cell, via conformational recognition

Aptamers are a class of short single-stranded DNA (ssDNA), RNA (ssRNA), or XNA (xeno nucleic acid, a synthetic nucleic acid analog) that can fit the targets with high affinity and specificity through the folding into diverse secondary and tertiary structures [16, 17] **(**Fig. 1**)**. The specificity of aptamer is comparable to antibody, so it is also named as chemical antibody due to the chemically synthesized production. Apart from that, aptamers own some unique advantages. Aptamer targets can range from ions [18], small molecules [19], peptides [20], RNA [21], proteins [22], cells [23], and even organs [24] because aptamers are generated by artificial selection and not necessary for immunogenicity to activate host immune system. Aptamers are generally chemically synthesized, making them easy to be modified and conjugated with other drugs or carriers [25]. Thus, aptamers have been widely studied in disease diagnosis, therapeutics, theragnostic, and biomarker discovery [26]. In this review, we focus on the updating application of different aptamer SELEX in biomarker discovery among various diseases, including cardiovascular diseases, cancers, neurodegeneration-related diseases, and others.

**Fig. 2:**
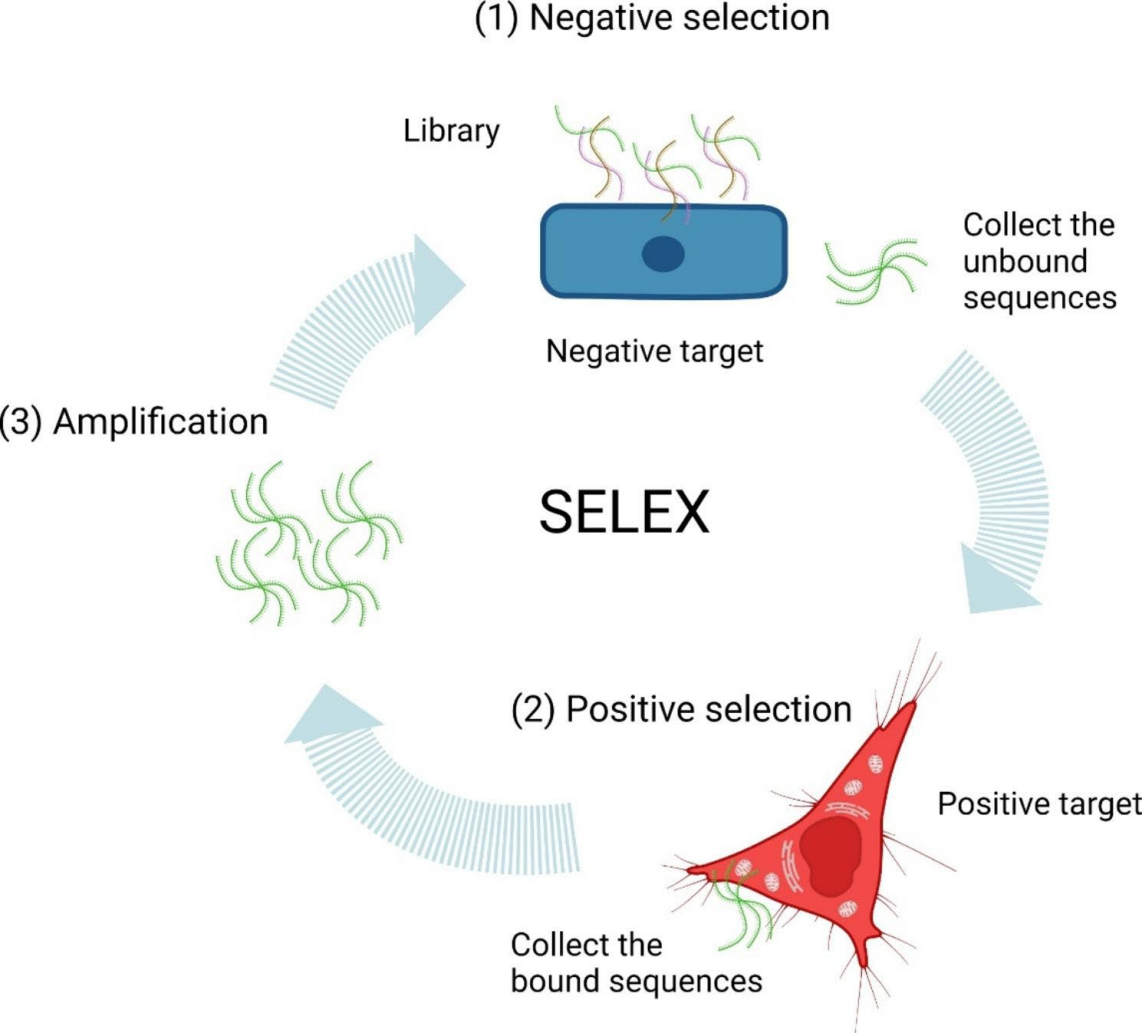
The general procedure of SELEX. (1) The library is incubated with a negative target, and the unbound sequences are collected; (2) The unbound sequences are incubated with a positive target, and the bound sequences ate collected; (3) The bound sequences are amplified for the next round of SELEX.

Systematic evolution of ligands through exponential enrichment (SELEX) is the primary technology method for generating aptamers that specifically bind to the targets [25]**(**Fig. 2**SELEX)**. Generally, DNA aptamer SELEX includes three major steps from the aptamer library. At the beginning of SELEX, the library needs to incubate with the negative target to remove the nonspecific binding sequences. The unbound sequences are collected for incubation with the positive target. These positive target binding sequences would be collected for PCR amplification, and the PCR product needs to be denatured into ssDNA for the next round of SELEX. Generally, 5 to 15 rounds of SELEX need to be performed during the complete SELEX procedure. Sequences of aptamers that bind to the target are obtained by sequencing. As to RNA-based aptamer SELEX, the RNA library is produced by transcription from DNA random library, which contains a promoter for RNA polymerase recognition. And the reverse transcription of target-binding RNA must be performed before PCR amplification. After amplification, the PCR-produced DNA is used as a template for RNA transcription, and this RNA aptamer pool would be put into the next round of SELEX. Except for the SELEX technique, the non-SELEX method is also developed for aptamer generation and mainly represents the no aptamer amplification involved in the selection [27]. The equilibrium capillary electrophoresis technology is utilized to separate the target-aptamer complex from the free DNA/RNA in the non-equilibrium capillary electrophoresis of equilibrium mixtures (NECEEM) [28]. By taking random nucleotide addition advantage of terminal deoxynucleotidyl transferase (TDT), scientists established a polynucleotide aptamer library for thrombin and lactoferrin aptamer selection [29]. Apart from aptamer selection, SOMAScan is an aptamer-based high-throughput proteomics assay designed to measure up to 7000 proteins simultaneously [30–32].

The major advantages of using aptamer as bait are its ease of synthesis and structural diversity. Libraries with structurally diverse aptamers can greatly improve the coverage in proteome analysis. Along with other advantages, such as being thermally stable and easy to be modified, the use of aptamers in biomarkers discovery has become more popular. In this review, the principle and the applications of SELEX-based and non-SELEX-based methods in biomarker discovery will be introduced. Meanwhile, in the second half of the review, how these aptamer-based methods facilitate biomarker discovery in cardiovascular diseases, cancer, neurodegenerative diseases and other diseases will be illustrated with examples.

## The technologies of aptamer-based biomarker discovery

By vitiating its sequence and structure, synthesized aptamer molecules can bind unlimited targets theoretically. Target-binding aptamer sequences are generated by SELEX, NECEEM, and SOMAScan in vitro. SELEX and SOMAScan can facilitate aptamer selection from multiple targets in complex samples, such as blood and cerebrospinal fluid (CSF), promoting biomarker discovery. Several variations on SELEX have been developed for detecting molecules from different components. Secretome SELEX was used for secreted protein biomarker discovery. Adaptive dynamic artificial poly-ligand targeting (ADAPT) was invented for exosome biomarker discovery. Cell-SELEX was developed for cell surface biomarker discovery. Tissue-SELEX was used for intracellular biomarker discovery. On top of the SELEX-based methods, non-SELEX-based methods like SOMAScan facilitated multiple proteins biomarker discovery.

### Secreted biomarker discovery through secretome SELEX

**Fig. 3:**
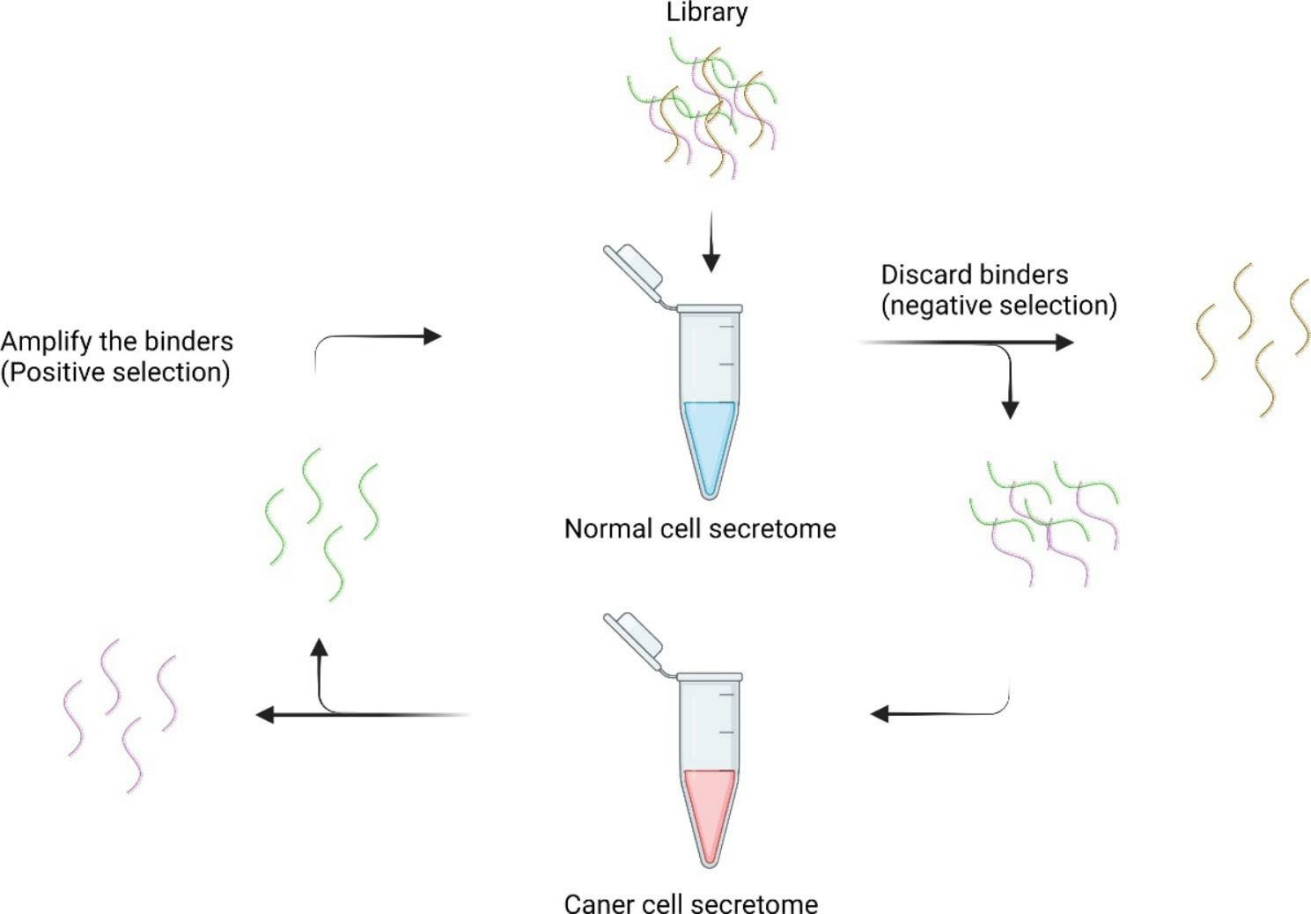
Scheme of secretome SELEX [34]. The initial library was first incubated with normal cell secretome. Aptamers that do not bind to the normal cell secretome were collected and proceeded to positive selection. After incubation with cancer cell secretome, bound aptamers were collected for further rounds of selection. After several rounds of selection, sequences of aptamers that bind to cancer cell secretome were analyzed by NGS.

Secretome, which refers to proteome from cell secretion or cell surface shedding, is a bonanza for disease biomarker discovery and in vitro diagnostics [33]. Secretome SELEX enables the identification of potential biomarkers and their binding aptamer from proteins secreted by a specific group of cells. Ray et al. successfully identified cyclophilin B (CypB) as a potential pancreatic cancer biomarker by analyzing secretomes from human pancreatic cancer cells [34]. The overall workflow is illustrated in Fig. 3. The RNA aptamer library was chemically modified with 2′-fluoropyrimidines (2′-Fluoro) to avoid nuclease degradation. The secretome from human pancreatic ductal adenocarcinoma cell MiaPaCa-2 was the positive target, and the secretory proteins from normal human pancreatic ductal epithelial cell HPDE-E6E7 were for negative selection. After nine rounds of selection, the RNA aptamer M9-5 was found to bind stronger to cancerous cell secretomes than non-cancerous cell secretomes. After separating the aptamer protein binding complex by gel electrophoresis, the MS analysis revealed cyclophilin B (CypB) as the target of M9-5. The ELISA assay and western blot results have further confirmed the binding of M9-5 to CypB. As a secreted protein in body fluids, CypB was reported to associate with several kinds of cancer progress as a tissue biomarker [35–40]. This secretome SELEX study first demonstrated the potential of CypB as a serum cancer biomarker for pancreatic cancer. Furthermore, aptamer M9-5 could be a potential diagnostic tool for early-stage pancreatic cancer in vitro diagnostics.

### Exosome biomarker discovery through ADAPT

**Fig. 4:**
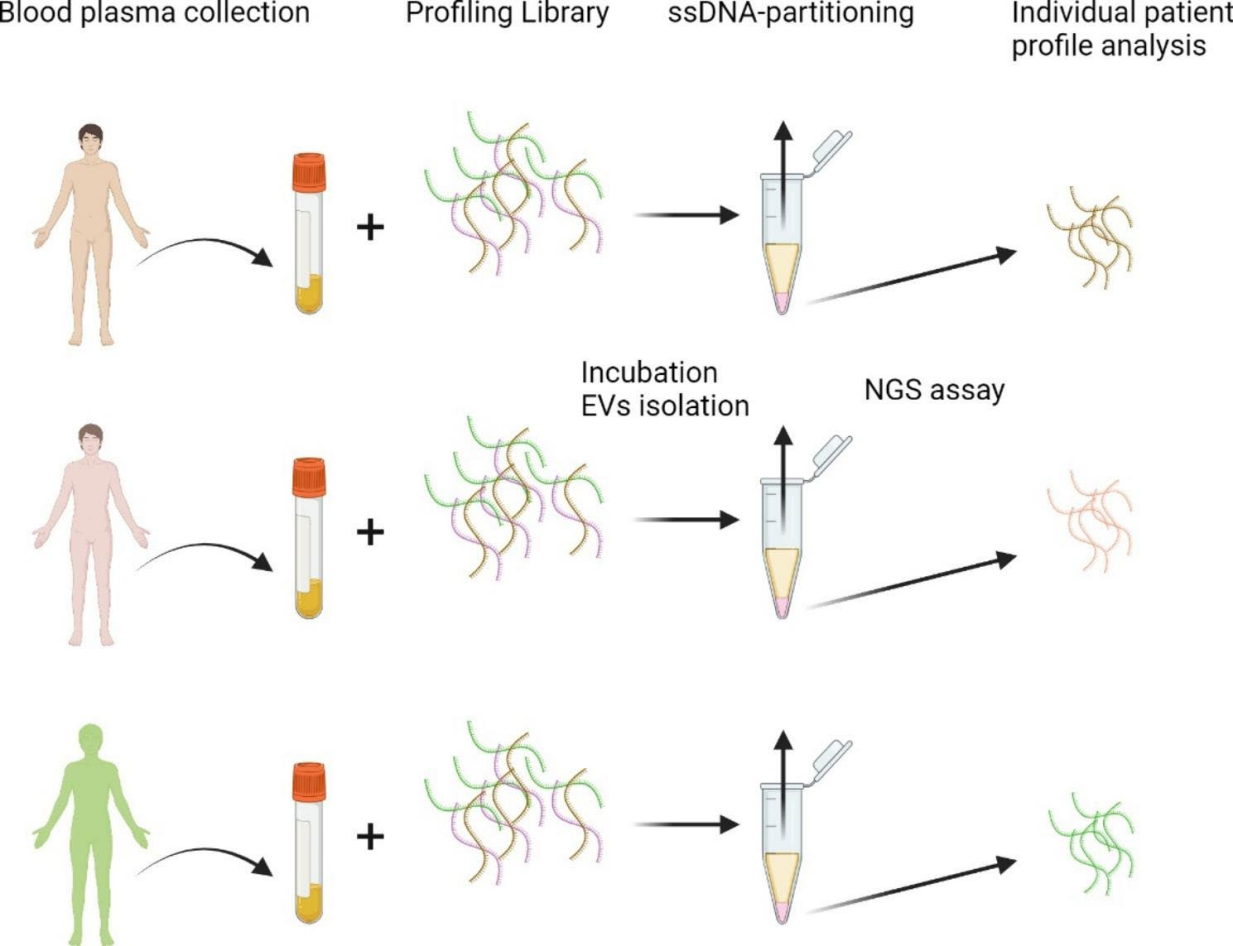
Workflow of Adaptive Dynamic Artificial Poly-ligand Targeting (ADAPT)[42]. The blood plasma samples are collected and incubated with the profiling library. Then the ssDNA-partitioning is performed for individual patient profile analysis

Exosomes are vesicles secreted by cells and contain biomolecules such as proteins and lipids. They participated in cancer progression by mediating intercellular communications and controlling various cancer processes such as metastasis, angiogenesis, and tumor microenvironment remodeling. Based on sample fractionation, Adaptive Dynamic Artificial Poly-ligand Targeting (ADAPT), a variant of SELEX [41], is able to identify and characterize subsets of biomolecules in liquid biopsies, such as blood plasma [42]. Domenyuk et al. have demonstrated the use of ADAPT to identify exosome biomarkers in breast cancer patients’ blood samples. Prior to ADAPT, an enriched aptamer library was needed to be prepared, which was generated from synthetic single-stranded DNA library L0. First, PCR-amplified L0 was either incubated with pooled blood plasma from breast cancer patients (positive target) or with pooled plasma from healthy women (negative target). In the following counter-selection step, the bound ssDNA from the positive target were retrieved and incubated with the negative target, and vice versa. The unbound ssDNA collected from the negative target was put back to be incubated with the positive target, and vice versa. Finally, the bound ssDNA from both the positive target and negative target were eluted and pooled together as a profiling library. The profiling library was amplified by PCR before undergoing the second and third rounds of enrichment. The profiling library from the final round was enriched with ssDNA bound to either a positive target or negative target, having improved specificity than the initial random library. In the ADAPT workflow **(**Fig. 4**)**, the enriched library was incubated with individual plasma samples. Fractionation was achieved by polyethylene glycol precipitation (PPT), in which exosomes in blood plasma were precipitated. Then, ssDNA was eluted for next-generation sequencing (NGS) analysis. Eventually, aptamer H1 and aptamer H11 were identified as the ligands of exosome-associated protein C1Q. C1Q plays a dual role in cancers, depending on the pathological mechanism of cancer [43]. It is a favorable prognostic factor for disease-free survival in basal-like breast cancer, while C1Q is a pro-tumorigenic factor in lung adenocarcinoma and clear cell renal cell carcinoma. Not only was C1Q identified as a potential candidate of breast cancer biomarkers, but also its binding conjugate aptamer H1 and H11 could further be developed for breast cancer diagnosis and prognosis. By incorporating fractionation, PPT, in this case, specific subpopulations of protein from blood samples can be analyzed. Blood sample collection is less invasive and requires less equipment, making disease screening easier. By analyzing exosomes in blood plasma, information of tumors at inaccessible locations can be obtained with less effort.

Another study from the University of Bonn also adopted ADAPT to develop biomarkers for subpopulations in prostate cancer [41]. The exosomes from vertebral cancer of the prostate (VCaP) and lymph node cancer of the prostate (LNCaP) were used in positive selection and negative selection, respectively. A total of five rounds of selection were performed. In round 1, only positive selection and PCR were performed. In rounds 2–5, positive selection followed by negative selection, as well as another positive selection was carried out before PCR. Sequencing and verification assay confirmed that sequence 7 showed specific affinity to VCaP cell exosome. Through pulldown assay, LC-MS/MS and western blot, Y-box binding protein 1 (YBX1) was identified as the binding protein of aptamer sequence 7. As a transcription regulator, YBX1 was reported to play multiple essential roles at the crossroads of the noncoding transcriptome, exosomal, and cytoplasmic granular signaling [44].

### Cell surface biomarker discovery through Cell-SELEX

Membrane proteins are essential for normal physiological processes and related to the disease’s pathological progression. Thus, it is of high importance in tumor imaging and targeted drug delivery [6]. Cell membrane molecular targets can be unveiled by targeting aptamers screened from Cell-SELEX technology. Distinguished from other SELEX methods, the whole living cells are directly utilized as the selection target in Cell-SELEX. Therefore, the cell surface molecules are displayed in a native folded state, which can further ameliorate the *in-situ* binding activity of generated aptamers [45] **(**Fig. 2**)**. Moreover, this technology provides an opportunity to develop aptamers for distinguishing normal and diseased cells, even though the exact different molecules between cells are unknown [46]. In addition, the identified aptamers from Cell-SELEX can facilitate fishing out the targeting molecules for new biomarkers discovery [47]. In the first place, the initial aptamer library was incubated with control cells for negative selection. And then, the unbound ssDNA needs to be incubated with target cells for positive selection. The bound ssDNA to target cells would be eluted and amplified by PCR to generate a new ssDNA pool for the following round of SELEX. Flow cytometer and fluorescence microscope could be used to monitor the screening progress. At last, sanger sequencing or NGS is used to resolve the sequence of candidate aptamer. The identified aptamers would selectively bind certain cell lines, such as between a cancer cell line and a normal cell line, or even between different subtypes of the cancer cell line, depending on the choice of positive target and negative target.

As early as 1998, Morris and Jensen performed cell-SELEX for the selection of membrane-binding aptamer [48]. The screened aptamers showed good binding affinity to the membrane of red blood cells. This was the first proof of concept for aptamers targeting a resemblance of biomolecules instead of a single protein. Later in 2003, aptamer GBI-10 was developed by Cell-SELEX, and it demonstrated selective binding to the glioblastoma cell line [49]. Based on a series of experiments, including affinity purification, protein electrophoresis, and LC-MS/MS, tenascin-C was recognized as the target of aptamer GBI-10. This was the first time for the identification of a specific protein biomarker using Cell-SELEX. Based on previous work, Tan’s group improved the protocol of Cell-SELEX by additional validation steps for binding site identification [50]. Five aptamers targeting CCRF-CEM cells were identified. To verify if the aptamers are targeting proteins on the cell surface, the binding affinity of these aptamers was tested with protease-K-treated cells. Three of them (sgc8, sgc3, and sgd3) lost binding affinity to protease-K-treated CCRF-CEM cells, while the other two aptamers (sgd2 and sgd4) were not affected. It was confirmed that sgc8, sgc3, and sgd3 were targeting cell surface proteins on CCRF-CEM cells. Berezovski et al. proposed Cell-SELEX can reveal the temporal expression of protein [51]. The SELEX was performed on immature and mature dendritic cells (iDCs and mDCs). A total of six proteins for iDCs and three for mDCs were identified as differentially expressed in dendritic cells. CD80 and CD40, previously known cell surface biomarkers for mDCs, were retrieved. Galectin 3, known to be upregulated in iDCs, was also identified as differentially expressed. Meanwhile, a number of proteins, such as lipoprotein lipase and sulfated glycoiprotein1, were found to be specifically expressed on the surface of iDCs for the first time. It should also be noted that a novel family of putative transmembrane, homologue of CXorf17, was observed. This study clearly demonstrated the strength of Cell-SELEX; it is not limited by fishing currently known protein only. Such findings may empower further research on dendritic cell labeling using aptamer-conjugate labels. Over the past two decades, Cell-SELEX has become a popular technique for identifying cell surface biomarkers for living cells [52].

### Intracellular biomarker discovery through Tissue-SELEX

**Fig. 5:**
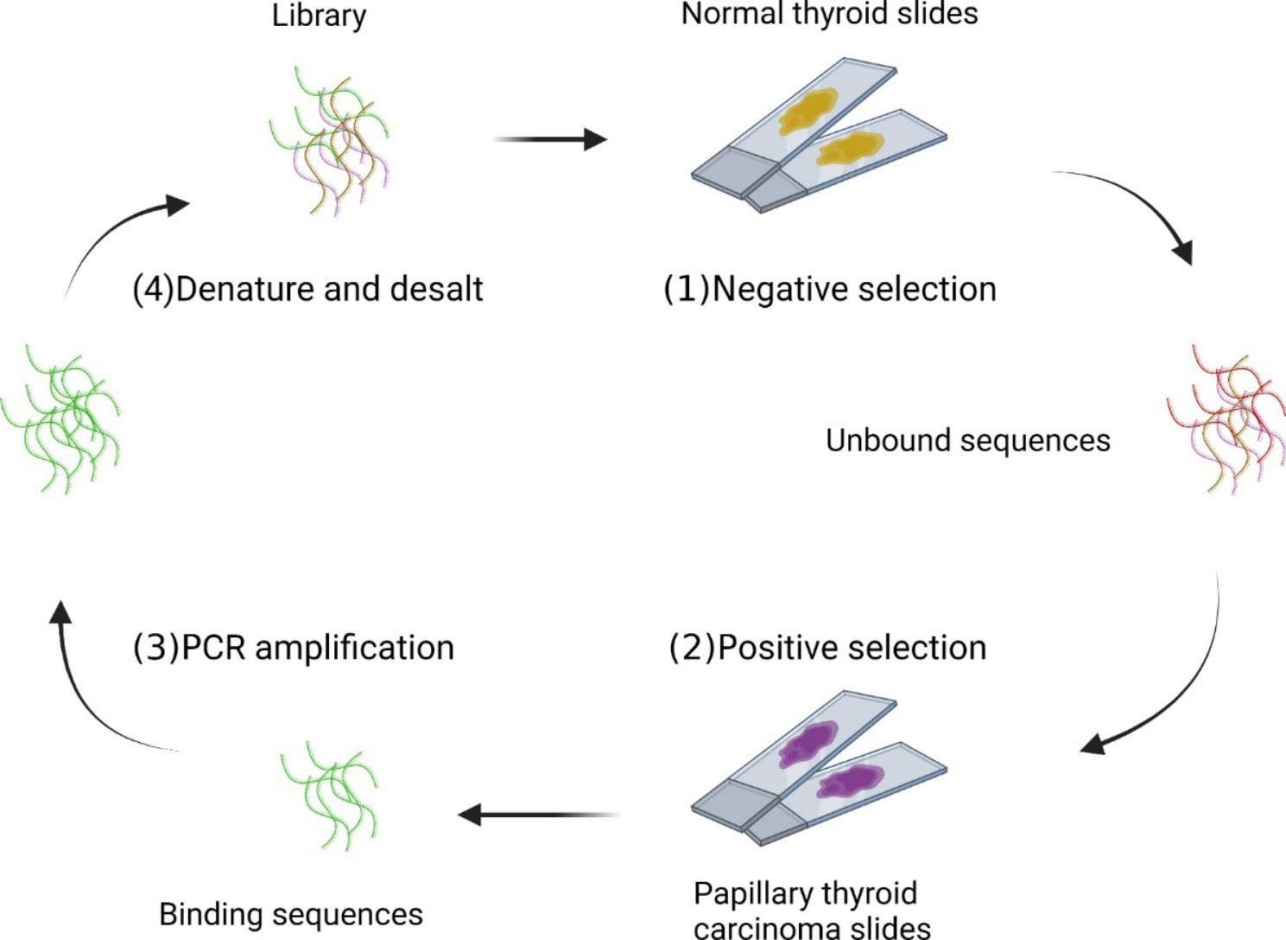
Illustration of tissue-SELEX procedure [55]. (1) The library is incubated with the negative target and the unbound sequences are collected; (2) The unbound sequences are incubated with the positive target and the bound sequences ate collected; (3) The bound sequences are amplified by PCR amplification; (4) The dsDNA of PCR product is denatured and desalted for next round of SELEX.

Apart from performing SELEX on living, intracellular biomarkers can be identified through SELEX on tissue samples. In situ tissue slide-based SELEX method was first proposed in 2009 for targeting the neoplastic tissues from breast cancer patients **(**Fig. 5**)** [53]. SELEX was directly performed on paraffin-embedded tissue slides containing samples from breast infiltrating ductal carcinoma and normal tissue adjacent to the carcinoma. Several adjustments were adapted to increase specificity. Counter-selection was included since the second round. Interaction time with positive targets was decreased gradually while the number of washes was increased gradually. After 12 rounds of selection, sequencing, and confocal imaging revealed specific binding of aptamer BC15 to breast cancer tissue slides. Interestingly, BC15 could still bind to the RNase-treated MCF7 cell nuclei, but the binding of BC15 to DNase-treated MCF7 cells was significantly enhanced. Therefore, it was concluded that BC15 may bind to a DNA-binding protein. By mass spectrometry, western blot and confocal analysis, heterogeneous nuclear ribonucleoprotein A1 was recognized as the target protein of aptamer BC15. In line with the finding, western blot and regular immunofluorescence revealed overexpression of hnRNP A1 in breast cancer. Subsequently, more studies unveiled the role of hnRNP A1 in tumor metastasis and cell invasion [54]. Another Tissue SELEX was performed on papillary thyroid carcinoma (PTC) with non-tumor thyroid as the negative target [55]. Aptamer TC-6 was identified to be targeting PTC tissues and TPC-1 cells specifically. Proteinase K and trypsin were used to digest the protein on the cell surface, but aptamer TC-6 still showed binding to the TPC-1 cells. Confocal fluorescent imaging showed localization of TC-6 in the cytoplasm, suggesting the uptake of aptamer by TPC 1 cells. In 2021, Wang’s group from Hunan University performed Tissue-SELEX between cancerous and adjacent normal liver tissue Sect. [56]. Aptamer SW1 was selected based on its specific binding to cancerous liver tissue and SMMC-7721 cells. Confocal fluorescent imaging demonstrated the co-localization of aptamer SW-1 and the nuclei. Tissue-SELEX preserves cell and extracellular components in near-native conditions, hence, enabling the discovery of biomarkers that may easily be degraded.

### Multiple biomarker discovery through SOMAScan Technology

**Fig. 6:**
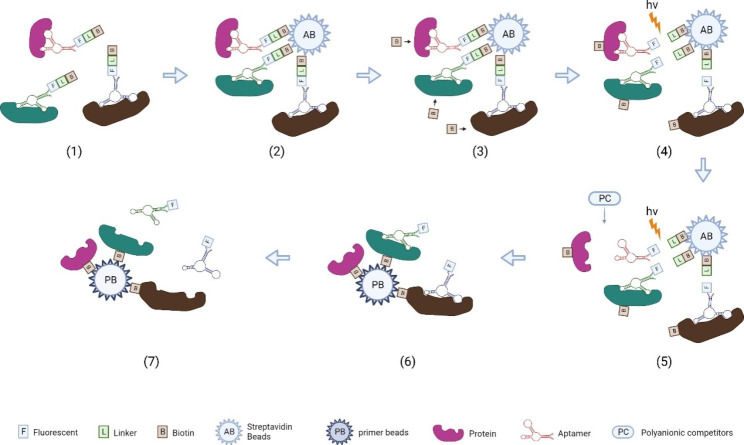
Schematic representation of SOMAScan. (1) The labeled SOMAmers are incubated with prepared samples to bind with proteins, and then the binding complexes are tagged with biotin (B) and fluorescent label (F); (2)The avidin beads (AB) are used to capture SOMAmers-protein complexes; (3)The biotin need to tag to the proteins; (4)The ultraviolet light (hv) is utilized to cleave the photocleavable group in order to release the complexes; (5) Polyanionic competitors (PC) are introduced to remove the nonspecific SOMAmers; (6)The primer bead (PB) facilitate the complexes capture; (7) Captured complexes are dissociated in specific high pH condition

SOMAScan is a multiplexed and highly sensitive proteomic analysis platform that can selectively detect thousands of proteins from complex biological samples **(**Fig. 6**)** [57, 58]. SOMAScan makes use of the properties of slow off-rate modified aptamers (SOMAmers) to distinguish strong binding complexes from nonspecific binding. Compared to conventional aptamer technology, the greatest strength of SOMAmers is the chemically modified nucleotide library which can improve the binding affinity with target proteins. Specifically, the chemical modification is induced at the 5’ position of dUTPs during library synthesis, aggrandizing the diversity of nucleic acids. Another strength of SOMAmers is the small dissociation constants and the two-step capture method. It minimizes nonspecific binding as well as cross-reaction. Generally, a SOMAmer contains a fluorescent tag at the 5’ position, and the tag links biotin with a photocleavable group. The SOMAmers library was incubated with protein samples, and the aptamer targets binding complexes were pulldown by streptavidin-coated beads. Then the biotins were labeled to the captured proteins, and the ultraviolet light was used to release the aptamer-protein complexes. In the following step, polyanionic competitors (PC) were involved in separating the binding between proteins and nonspecific aptamers. In the next step, the specific aptamer-protein complexes were captured by complementary oligonucleotide primer beads. Finally, the complexes were separated by high pH solutions, and the harvested proteins could be further analyzed by denaturing polyacrylamide gel electrophoresis (PAGE) and MS [57].

SOMAScan has been applied in the biomarker discovery of various diseases [57, 58]. By analyzing the plasma of chronic kidney disease patients through SOMAScan, 58 proteins, such as β2-microglobulin, Pleotrophin, Angiopoietin-2, and Cystatin C were successfully identified as potential biomarkers [57]. Ostroff et al. have developed a panel of 12 proteins that can detect early-stage non-small cell lung cancer with good sensitivity and specificity [58]. In 2012, Baird et al. performed SOMAScan on CSF for the first time [59]. Two hundred forty-eight proteins were found to be upregulated in Parkinson’s disease, including proteins involved in inflammation and injury response. This study provides new insight into age-related changes in CSF proteome. Webber and colleagues uncovered 300 novel potential protein biomarkers for prostate cancers by SOMAScan [60].

## The application of aptamer-based biomarker discovery in various diseases

Biomarkers are of great importance in disease diagnosis and therapeutics. By discovering biomarkers for an early stage of disease, interventions can be implemented during the ideal therapeutic window period, improving disease survival. While most traditional therapeutics come with serious side effects, biomarker-targeted therapy precisely acts on the diseased cells with improved efficacy and fewer side effects. Here, we will discuss how aptamer-based methods accelerate biomarker discovery in a range of diseases.

### Cardiovascular Diseases

Myocardial infarction (MI) occurs when blood flow to the myocardium decreases or ceases as a result of thrombosis or vascular occlusion. It is the leading cause of morbidity and mortality among all cardiovascular diseases [61, 62]. The first SOMAScan for biomarker screening of planned myocardial infarction (PMI), which resemble the clinical features of spontaneous MI, was reported in 2016 [63]. Two hundred seventeen proteins were identified with significant change within 24 h post-injury. Some of these proteins, including malate dehydrogenase, cytoplasmic (MDHC), triosephosphate isomerase (TPI), leukocyte inhibitory factor soluble receptor (LIFsr), and fibroblast growth factor-18 (FGF18) were first shown to be associated with MI. Analysis of 1310 serum proteins in patients with acute myocardial infarction (AMI) was performed through SOMAScan [64]. The relative concentration of 35 proteins showed differential expressions. HAMP and FGG were aberrantly expressed exclusively in AMI. After measuring 1129 proteins of samples from patients undergoing septal alcohol ablation for hypertrophic cardiomyopathy, 247 proteins were observed with significant differences in expression level. Many of them were intracellular proteins [65]. By employing slow off-rate aptamers, 1074 circulating proteins were analyzed from an MI cohort [66]. Supplemented With enzyme immunoassay, fives proteins (LPB, HAMP, IGFBP4, CCL23, PRTN3) were confirmed as novel biomarkers of MI. More interestingly, the administration of tocilizumab, an anti-IL-6 antibody, significantly altered the expression pattern of these five proteins, implying their potential to monitor treatment response.

Heart failure (HF) has become society’s health burden and epidemic disease in the long-life-span modern world [67]. Heart failure is caused by multiple factors that a single biomarker may not be sufficient for diagnosis. Thus, m aptamer-based biomarker discovery outshines traditional antibody-based assays in terms of coverage. Plasma samples from 412 HF patients, 571 no HF patients and 332 no HF or cardiovascular disease patients were analyzed by DNA aptamer-based proteomic platform [68]. Nine protein candidates were identified among 1129 proteins analyzed. Two of them, angiopoietin-2 (ANG-2) and thrombospondin-2 (TSP-2) were revealed to be related to HF after three separate validation cohorts. Apolipoprotein M (APOM) plays multiple roles in pleiotropic effects, such as anti-inflammatory effects, antioxidant and anti-atherogenic effects [69, 70]. APOM increase cardiomyocyte survival by enhancing nitric oxide production and vasodilation in animal models [71, 72]. The relevance of APOM in human HF was investigated by researchers from the University of Pennsylvania [73]. After analyzing around 5000 proteins from Penn-HF study participants, a few canonical pathways were found to be associated with APOM, such as inflammation, coagulation system and LXR/RXR activation. NT-proBNP, THBS-2, and MBL were recognized as the higher risk factors in HF. Conversely, EGFR, GDF11/8, and hemojuvelin were found to associate with lower risk in another SOMAScan study [74].

Cardiac hypertrophy (CH) refers to the thickening of heart muscle in response to the increased workload of the heart [75]. In the study of Francesco and colleagues in 2013, they used surgical techniques to let different ages of animals share circulation, and the cardiac hypertrophy in old mice regressed dramatically [76]. In addition, modified aptamer-based proteomics was utilized to explore the youth factor, and TGF-β superfamily member GDF11 was recognized as a circulating factor for declining age. Moreover, the restoring GDF11 in old mice models exhibited therapeutic effects of parabiosis and reversing age-related hypertrophy. SOMAScan facilitated serum proteomic analysis of an age-stratified healthy group, and further community-based group comparing with HF patients has been executed to identify biomarkers for pathological cardiac hypertrophy and heart failure [77]. The level of circulation C-C motif chemokine ligand 17 (CCL17) increased with age and correlated to CH. *Ccl17* knock-out animal model revealed repression of aging, angiotensin II (Ang II)-induced CH and fibrosis. Apart from that, anti-CCL17 neutralizing antibody inhibited Ang II related pathological cardiac remodeling. This supports CCL17 as a potential therapeutic target for age-related and Ang II-related pathological CH and HF.

Acquired aplastic anemia (AA) is a rare but life-threatening bone marrow hematopoietic failure syndrome because of pancytopenia and BM hypocellularity [78, 79]. In order to identify new protein biomarkers for evaluation of response to immunosuppressive therapies, SOMAScan was applied to analyze serum proteome and plasma proteome, before and after treatment. SOMAScan was applied to measure 1141 proteins from the serum of 28 AA patients before and after treatment, as 1317 proteins from plasma of 7 severe AA patients remedied for standard IST and a thrombopoietin receptor agonist [80]. Their report uncovered 19 candidate diagnostic and prognostic biomarkers from the serums, comparing 28 novel proteins from the plasmas. The immunobead-based multiplex assay was conducted to verify potential biomarkers further. Four biomarkers were confirmed to be associated with AA diagnosis and long-term response to IST.

### Cancers

Today, cancers have become the second deadliest disease after heart ailments [62, 81]. Cancer is defined by uncontrollable cell growth and division. It can be attributed to gene mutation, exposure to radiation and chemicals, and pathogenic infections [82]. During the last decades, the overall survival rate, progression-free survival rate, and the life quality of cancer patients have improved dramatically because of biotechnological breakthroughs [83]. Cancer biomarkers are molecules that indicate the presence of cancer, its location, and the stage of the disease. Cancer biomarkers can be found in blood, urine, or tissues of cancer patients. The detection of cancer biomarkers can help diagnose cancer at an early stage, monitor the response to cancer treatment, and predict the prognosis or likelihood of recurrence. Biomarkers are also crucial for cancer research, enabling the identification of new cancer drug targets. The current developments in precision medicine rely heavily on the mechanisms-, data- and technology-driven biomarker discovery [84].

Through the progress in the research and therapy of T cell acute lymphoblastic leukemia (ALL) has been ameliorated over the past four decades, the improving the outcome of T cell ALL has been demonstrated with more difficulty and has depended on modifications of standard chemotherapies (high-dose cytarabine, high-dose methotrexate, asparaginase, nelarabine) [85]. And the application of newly emerged Chimeric antigen receptor T-cell therapy (CAR-T) to T-cells was challenged as well, owing to the limited ability to distinguish between therapeutic, normal, and malignant T-cells [86]. Cell-SELEX was utilized by Shangguan et al. for the biomarker discovery among a series of leukemia cell lines, and the MS result suggested protein tyrosine kinase 7 (PTK7) as the specific biomarker candidate for T-cell ALL [87].

Nasopharyngeal carcinoma (NPC) is EBV-associated head and neck squamous cell carcinoma [88]. Although the 5-year survival rate of NPC is over 60% based on the statistical data from the American Cancer Society [89], the high incidence of local recurrence or metastasis rate still threatens the patients’ health [90]. By using Cell-SELEX, four aptamers (S3, S5, S12, and S27) were selected with specific affinity to the NPC cell line (NPC 5-8 F) but not to the normal nasopharyngeal (NP) cell line (NP69) [91]. Further investigation demonstrated cell membrane protein CD109 as the target of aptamer S3. Overexpression of CD109 has also been detected in NPC stem-like cells. Moreover, recent studies have revealed that CD109 is overexpressed in multiple human cancers, including squamous cell carcinoma of the lung [92], vulva [93] and uterine cervix [94], adenosquamous carcinoma of the gallbladder [95], and ductal adenocarcinoma of the pancreas [96]. Recently, a meta-analysis has proved the association between the high level of CD109 expression and poor overall survival in cancer patients, suggesting the use of CD109 as a prognostic [97].

Under the current front-line standard of care, the five-year survival rate of ovarian cancer is as low as 45%[98]. Biomarkers are desperately needed for early diagnosis and targeted therapy. As early as 2010, the Cell-SELEX was performed on ovarian cell lines (TOV-21G and CAOV-3) [99]. A series of aptamers have been obtained, but the targets of these aptamers have still waited for investigation. After years of efforts, Van Simaeys et al. identified stress-induced phosphoprotein 1 (STIP1) as the targeting protein of TOV6, an aptamer that targets ovarian cell line TOV-21G [60]. A serial assay, including MS, flow cytometry, siRNA silence, and protein blotting, validated stress-induced phosphoprotein 1 (STIP1) as the targeting protein. And the blocking of STIP1 by TOV6 inhibited the invasion of TOV-21G cells. Aptamer HF3 and aptamer HA5 have been generated by Cell-SELEX for targeting the paclitaxel-resistant ovarian cell line, A2780T [100]. During protein glycosylation, tunicamycin can inhibit the transfer of saccharide moieties to dolichol during dolichol-linked glycoprotein synthesis [101]. Additionally, wheat germ agglutinin (WGA) can specifically recognize sugar and facilitate the detection of glycoproteins [102]. So, the tunicamycin-treated A2780T cells decreased the glycoproteins expression as well as the binding fluorescence intensity of fluorescein-conjugated WGA. More interestingly, the aptamer HF3 and HA5 binding affinity to tunicamycin treatment A2780T cells was also decreased significantly, which indicated the targets of these two aptamers were glycoproteins on the cellular membrane. After the combination of aptamer-based SOMAscan and antibody-based Olink platform proximity extension assay (PEA), proteomics between ovarian carcinoma recurrence and proteins released into the tumor microenvironment have been profiled [103]. Three proteins (HSPA1A, BCAM, and CTSZ) play as the indicators of a short relapse-free survival (RFS), and six intracellular proteins (LCK, MAPK14 (p38), STK17B (DRAK2), CAMK2B and CAMK2D) are strongly associated with longer RFS. Another multiplatform affinity proteomics (PEA, SOMAScan, and ELISA) study in metastasis and immune suppression in ovarian cancer plasma identified SPINT2 gene expression associated with an increased survival rate [104].

Pancreatic cancer has a high mortality rate owing to the late diagnosis and resistance to chemotherapy [105]. As previously mentioned, secretome SELEX identified CypB, which could be a promising biomarker for the early detection of pancreatic cancer [34]. Cell-SELEX-generated aptamer SQ-2 was found with a specific binding affinity to pancreatic cancer cells by targeting alkaline phosphatase placental-like 2 (ALPPL-2) [106]. On top of cell membrane localization, ALPPL-2 was detected as a secretory protein from pancreatic cells. This expanded the potential of ALPPL-2 as a diagnostic biomarker. A DNA aptamer XQ-2d was developed by Cell-SELEX in 2015 that showed selectivity to pancreatic cancer cell line PL45[107]. Later in 2017, CD71 was identified as the binding target of aptamer XQ-2D by MS and flowcytometry [108]. RNA P15 was also fished out from the library by Cell-SELEX. LC-MS/MS revealed RNA P15 bound to intermediate filament vimentin, which is associated with cancer cell metastasis [109]. Similarly, 14 rounds of RNA aptamer Cell-SELEX have been conducted on target pancreatic cancer cells [110]. This study unleashed the potential of aptamer P19 and P1 for drug delivery. Through imaging mass cytometry (IMC) and MS, mortalin was confirmed as the target of aptamer P19 and P1[111]. Mortalin was aberrantly expressed in pancreatic cancer patients with low survival rates, which revealed its potential for prognostic prediction. Besides SELEX, SOMAScan-facilitated biomarker discovery for patients suffering from pancreatic ductal adenocarcinoma (PDAC) and cachexia was online at the end of 2020[112]. Four proteins were found common for local/cachexia (C1R, PRKCG, ELANE, SOST: all oppositely regulated) and another four proteins associated with metastatic/cachexia (SERPINA6, PDGFRA, PRSS2, PRSS1: all consistently changed), which suggested the stage and cachexia status might be molecularly independent.

The five years survival rate of lung cancer patients only researched 20%, which has become the world’s most deadly form of cancer [113, 114]. Like other cancers, symptoms of early-stage lung cancer may not be noticeable or may be mistaken as an infection. Patients may have missed the ideal therapeutic window due to a delay in diagnosis. Lung cancer can be further classified into three main types, non-small cell lung cancer, adenocarcinoma and squamous cell carcinoma. Each subtype of lung cancer differs in pathophysiology and response to treatment. Thus, full characterization with biomarkers discovery is needed for various subtypes of lung cancer. As early as 2008, the first Cell-SELEX research for targeting lung cancer cells was reported [115]. However, no novel protein biomarker was revealed till the first tissue-SELEX of lung adenocarcinoma study in 2015[116]. Four aptamers showed specific binding to lung adenocarcinoma cells but not to non-small-cell lung cancer (NSCLC) cell line A549. MS revealed candidate biomarkers cathepsin D, vimentin, annexin A5, annexin A2, histone H2B and clusterin. A novel small-cell lung cancer biomarker was identified by Cell-SELEX-generated aptamers in 2019, and the target of aptamer C12 was high density lipoprotein binding protein (HDLBP)[117]. Researchers were then interested in the role of HDLBP in small-cell lung cancer. After HDLBP knockdown by siRNA, proliferation and metastasis of SCLC cells were inhibited in vitro, and tumor formation in vivo was decelerated. Different from secretome SELEX, serum-SELEX needs to link serum proteins to a carboxylate-modified magnetic bead for the separation of unbound sequences. In the report of Zhao et al., CLEC3B was identified as the target protein by MALDI-TOF MS and secondary peptide sequencing MS analysis [118]. In addition, the ELISA confirmed the overexpression of CLEC3B in lung cancer patients, which makes it useful in the diagnosis [119]. Aptamer AP-9R was developed by Cell-SELEX for targeting cancer stem cells of lung cancer, and the target protein is revealed as annexin A2. It was recognized as an unfavorable marker in renal, pancreatic, liver, urothelial, and lung cancer [120]. In order to discover blood protein biomarkers in NSCLC, researchers from SomaLogic analyzed 813 proteins of 1,035 asymptomatic participants through SOMAScan in 2010. A 12-protein panel (cadherin-1, CD30 ligand, endostatin, HSP90α, LRIG3, MIP-4, pleiotrophin, PRKCI, RGM-C, SCF-sR, sL-selectin, and YES) were developed for improved diagnosis and prognosis [58]. In the same year, another group discovered the protein signature of lung cancer tissues study through SOMAScan. A list of tissue biomarkers was found to be associated with angiogenesis, inflammation & apoptosis, invasion, metastasis, growth and metabolism [121].

Glioblastoma is the most aggressive type and most common type of brain cancer. Isocitrate dehydrogenase (IDH)-wildtype glioblastoma account for more than 90% of the cases. Common cellular signaling pathways directed in targeted therapies have failed to improve outcomes in glioblastoma, including phosphoinositide 3-kinase (PI3K)/protein kinase B (AKT)/mammalian target of rapamycin (mTOR), p53 and the retinoblastoma (RB) pathway, or epidermal growth factor receptor (EGFR) gene amplification or mutation [122]. Novel biomarkers for diagnoses and targeted therapy are desirable. Cell-SELEX for glioblastoma biomarker discovery was first reported in 2003. Tenascin C, an oligomeric glycoprotein involved in embryogenesis and oncogenesis, was identified as the target of aptamer GBI-10 [47]. SOMAScan revealed abnormal expression levels in CKM (creatine kinase, isoform M), MDK (midkine), FN1 (fibronectin 1), STAT6, STAT1, and B-cell factor CD59[123].

### Neurodegeneration-related Diseases

Parkinson’s disease (PD) is a common progressive neurodegenerative disease. Meanwhile, 5 million patients worldwide are suffering from tremors and slow movement, and there are no disease-modifying therapies available [124]. As a specimen of the brain is extremely difficult to collect, reliable and accessible blood-based biomarkers for diagnosis and prognosis are urgently needed. After analysis of 1,129 proteins from 141 plasma samples by SOMAScan, it was found that BSP, OMD, ACY1, and GHR in serum were associated with PD [124]. In another study where SOMAScan was employed to filter over 1300 proteins from 85 PD patients and 93 healthy controls, 14 differentially expressed proteins were confirmed as stable potential diagnostic markers. Additionally, ALCAM, contactin 1, CD36, DUS3, NEGR1, Notch1, TrkB, and BTK were obviously correlated with longitudinal clinical scores, implicating the potential to predict the progression of various PD phenotypes [125].

Multiple sclerosis can cause acute inflammatory lesions and chronic inflammation in the central nervous system (CNS), resulting in tissue damage and disability [126]. After analyzing extracellular vehicles in CSF through SOMAScan, 50 proteins out of 1128 were particularly enriched in multiple sclerosis vesicles, such as KLKB1, ApoE4, DKK3, C6 and S100A9 [127]. Bioinformatics analysis suggested these proteins were correlated to complement pathway, coagulation and wound healing. Masvekar group used SOMAScan to test the CSF vehicles from 431 patients suffering from neuroimmune diseases and healthy participants, then over 1000 proteins were detected. Finally, astrocyte cluster 8 (MMP7, SERPINA3, GZMA, and CLIC1) and microglial cluster 2 (DSG2 and TNFRSF25) were elevated in multiple sclerosis patients and suggested the pathogenic role of these biomarkers during multiple sclerosis progression [128].

### Other diseases

In addition to chronic conditions, biomarkers could also aid in diagnosis and treatment evaluation in infectious diseases. Pulmonary tuberculosis (TB) is caused by Mycobacterium tuberculosis infection. It has become one of the most significant health threats in the world, leading to over 1.6 million deaths in 2021[129]. With the long treatment duration resulting in increasing rates of drug resistance, it is necessary to discover novel biomarkers for new drug development as well as new prognosis factor exploration. The application of SOMAScan to compare the proteomic difference between the serum of 39 PTB patients before and after eight weeks of treatment was executed and reported in 2013[130]. A series of proteins expression levels has been confirmed with significant differences after treatment, such as TSP4, TIMP-2, SEPR, MRC-2, Antithrombin III, SAA, CRP, NPS-PLA2, LEAP-1, and LBP. It provided new understanding insight for treatment and subsequent healing processes as the response marker for effective therapies. Another in-depth proteomic 4,000-plex SOMAscan assay was performed for 1,470 serum samples analysis from seven TB endemic countries [131]. SAA (serum amyloid protein A), NPS-PLA2 (secreted phospholipase A2), and CA6 (carbonic anhydrase 6) demonstrated the largest median fold changes for non-sputum biomarker tests to diagnose active TB.

Rheumatoid arthritis (RA) is one of the most prevalent chronic systemic autoimmune diseases [132]. Although RA is primarily characterized by synovitis of the joints, many extra-articular manifestations (EMs) and comorbidities occur because of the complex, chronic, inflammatory, and autoimmune nature of RA [133]. Murota et al. analyze serum proteins from RA patients, people who have Sjogren’s syndrome, and healthy individuals using SOMAScan assay [134]. Interleukin 16 was considered to be a potential biomarker implicating clinical response for early treatment of RA. Another combinational study of LC-MS/MS, Luminex xMAP, and SOMAScan was used to discover unambiguous biomarkers for psoriatic arthritis (PsA) and RA. One hundred seventy-two proteins were identified with differential expression, in which 42 proteins were revealed by LC-MS/MS, 3 proteins through Luminex xMAP and 127 proteins owed to SOMAScan [135] [136]. Similarly, to evaluate the different serum protein biomarkers between PsA and RA, which may facilitate the appropriate early intervention, nano-liquid chromatography mass spectrometry (nano-LC-MS/MS), SomaScan and Luminex were combined utilization. Moreover, the use of multivariate machine learning presents a serum protein biomarker panel that could distinguish the patients with early-onset IA with PsA from those with RA. Liam J O’Neil and colleagues studied the association of serum proteomic characters during RA development [137]. SomaScan technology facilitated the evaluation of the protein levels in multiple longitudinal serum samples from 17 participants, and machine learning was utilized to uncover the protein signature for the highest risk of future RA development. These examples clearly demonstrated the robustness of SOMAScan in biomarker discovery. Proteins interact with each other in many cellular processes. Proteins with elevated expression in a diseased state may not necessarily be involved in pathogenesis. Combining SOMAScan and network analysis of proteins would reduce the false positive rate.

Acute kidney injury (AKI) is characterized by the sudden loss of excretory kidney function, leading to high mortality. Serum biomarkers may be beneficial to the understanding of the pathophysiological processes [138]. After profiling as many as 1305 proteins from serum samples on day one and day eight after onset, higher serum levels of FGF23, tPA, MMP8, and suPAR were related to higher mortality [139]. Another SOMAScan study revealed that higher serum levels of CXCL11, Wnt-7a, BTK, c-Myc, CXCL2/CXCL3, TIMP-3, CCL5, CD86, ghrelin, PDGF-C, survivin, IL-9, EGF, CA2, and neuregulin-1, and lower levels of soluble CXCL16, FGF23, IL-6, IL1RL1, and stanniocalcin-1 were significantly related to better kidney recovery [140]. This research work identified new potential biomarkers for the prognosis of AKI. With the development of SOMAScan technology, up to 7000 proteins can be quantified per biosample, and then Richard X. Liu et al. analyzed 5,284 proteins from the biosamples of 294 patients [32]. After a combination comparison with immunoassays, they revealed a strong correlation between inter-platform operability and higher biomarker concentrations.

Duchenne muscular dystrophy (DMD) is a severe, progressive hereditary disease correlated to the X chromosome. Patients suffering from DMD will experience muscle loss and movement dysfunction [141]. SOMAScan analyzed 1125 serum proteins from DMD patients and healthy controls [142]. Forty-four differentially expressed proteins were identified. The concentration of these proteins was found to be age-dependent, suggesting their potential as novel diagnostic and therapeutic targets. Glucocorticoid-responsive serum protein biomarkers for DMD have also been evaluated by SOMAScan.17 serum protein levels were shown to be associated with DMD. As the concentration of these proteins tended to normalize with the glucocorticoid treatment, they could serve as indicators for evaluating treatment response [142]. Baseline clinical severity related serum protein biomarkers were quantified in the young male patients with steroid-naïve DMD using SOMAscan® aptamer profiling, and three proteins were strongly associated with predicting clinical and histological severity [143]. Reduction of Erb-B2 receptor tyrosine kinase 4 (ERBB4) and ciliary neurotrophic factor (CNTF) in blood and muscle accompanied by increasing severity. The reduction of superoxide dismutase1 in muscle and an increase in blood related to more severe symptoms. Serum pharmacodynamic biomarkers were analyzed to compare the treatment of DMD between prednisone and deflazacort [144]. Longitudinal trajectories of SOMAscan® protein data revealed that seventeen candidates were significantly altered between the two drugs. In addition, the cross-validation with ELISA confirmed IGFBP-2 related to diabetes complications and MMP-3 related to extracellular remodeling. These proteins could serve as biomarkers for DMD prognosis.

Idiopathic pulmonary fibrosis (IPF) is a chronic progressive disease with unclear etiological factors. Manifestations usually involve relentless scarring in the lung parenchyma, resulting in lower life quality and even earlier mortality [145]. In the study by Todd et al., serum proteome was compared between 300 patients with IPF and 100 participants without known lung disease through SOMAScan. Five hundred forty-one proteins with differential expression were identified [146]. Among these candidate biomarkers, 14 proteins were classified to associate with forced vital capacity (FVC) % predicted, 23 with diffusion capacity of the lung for carbon monoxide (DLco) % % predicted, and 14 with composite physiologic index (CPI). However, only four proteins, including roundabout homolog-2, spondin-1, polymeric immunoglobulin receptor (PIGR), and intercellular adhesion molecule-5 were positively correlated to severity measures of all three diseases. To further explore more reliable biomarkers of IPF, integrated analysis for plasma proteomics and lung transcriptomics has been conducted by Sivakumar et al.[147]. Mucosal (CCL25 and CCL28) and Th2 (CCL17 and CCL22) chemokines were significantly higher in expression levels in IPF patients. As the mast cell maturation chemokine, CXCL12 was detected with an aberrantly higher level in lung plasma. This panel of tissue and circulating biomarkers can be explored for diagnosis, therapy, and prognosis in IPF.

## Discussion

As a measurable substance or characteristic in an organism that can indicate the presence or severity of a disease, infection, or other physiological state, biomarkers have constantly expanded their influence in modern medicine [148]. Biomarkers are essential in improving disease diagnosis, prognosis, and treatment by providing a way to monitor a patient’s response to therapy. They also play a critical role in drug discovery and development, allowing for identifying and validating potential drug targets. Owe to modern biotechnology exploration, such as the development of mass-spectrometry, molecular biology, omics study, and bioinformatics, the biomarkers discovery has been well established. For instance, the protein biomarker discovery workflow in the proteomic study includes sample collection, sample preparation, liquid chromatography, ion mobility, MS1, peptide fragmentation, MS2, peptide/protein identification, protein quantification, and bioinformatic analysis. Additionally, artificial intelligence (AI), such as machine learning and deep learning, can facilitate data generation [149]. The combination use of omics research, mechanism-based biomarker discovery, and AI facilitation have promoted biomarker discovery to a new and high stage. And all these efforts are to separate and quantify the potential specific molecules between disease groups and control groups. Aptamer-based methods can facilitate the potential biomarkers concentrating at the beginning, which lowers the technical difficulty as well. Moreover, the flexible SELEX workflow and high throughput SOMAScan technology can adapt to various application scenarios, regardless of the body fluid, cell line, or tissue samples. As summarized in this review, aptamer-based technologies generated biomarkers have been verified as predictive, diagnostic, or prognostic tools in kinds of diseases, including cardiovascular diseases, cancers, neurodegeneration-related diseases and so on.

Undeniably, there are also some challenges to aptamer-based biomarker identification, including rigorous standards for aptamer application, inefficient protein extraction, vague criteria for biomarker validation, and low clinical translation rate [150]. These obstacles are gradually being overcome because of the continuous advance of aptamer-based technologies and the combination of usage with other biomarker discovery platforms, such as ELISA, transcriptome analysis, Luminex xMAP, etc. Furthermore, the newly emerged AI technology has become an integral component of the proteomics enterprise [149], and machine learning (ML) has also been applied in the SOMAScan method [151], which may reveal and illustrate the mechanism between biomarkers and disease progress more clearly. Applying aptamer in biomarker discovery can explore new protein biomarkers, from the body fluid, secretome, EVs, and cell surface to intercellular apart. Plenty of studies have revealed various biomarkers discovery based on aptamer facilitated strategy in order to evaluate different diseases’ risk prediction, diagnosis, and prognosis, including cardiovascular diseases, cancers, neurodegeneration-related diseases and so on. These studies not only proved the feasibility of utilizing several SELEX methods and SOMAScan in protein biomarker selection but also demonstrated the advantages of taking novel biomarker panels in disease progression assessment. With the advance in aptamer-based technology and combination with other platforms, we hope to see more biomarkers being put into clinical use.

## Data Availability

Not applicable.

## List of abbreviations

SELEX: Systematic Evolution of Ligands by Exponential Enrichment
SomaScan: Slow Off-Rate Modified Aptamers Scan
HER2: Human epidermal growth factor receptor 2
MS: Mass spectrometry
HPLC: High-Performance Liquid Chromatography
ELISA: Enzyme-linked immunosorbent assay
PCR: Polymerase chain reaction
NECEEM: Non-equilibrium capillary electrophoresis of equilibrium mixtures
TDT: Terminal deoxynucleotidyl transferase
ADAPT: Adaptive dynamic artificial poly-ligand targeting
CypB: Cyclophilin B
NGS: Next-generation sequencing
VCaP: Vertebral cancer of the prostate
LNCaP: Lymph node cancer of the prostate
YBX1: Y-box binding protein 1
DC: Dendritic cell
PTC: Papillary thyroid carcinoma
PAGE: Polyacrylamide gel electrophoresis
NSCLC: Non-small cell lung cancer
MDHC: Malate dehydrogenase cytoplasmic
TPI: Triosephosphate isomerase
LIFsr: Leukocyte inhibitory factor soluble receptor
AMI: Acute myocardial infarction
HF: Heart failure
CVD: Cardiovascular disease
ANG-2: Angiopoietin-2
TSP-2: Thrombospondin-2
APOM: Apolipoprotein M
CH: Cardiac hypertrophy
CCL17: C-C motif chemokine ligand 17
AA: Aplastic anemia
ALL: Acute lymphoblastic leukemia
CAR-T: Chimeric antigen receptor T-cell therapy
PTK7: Protein tyrosine kinase 7
NPC: Nasopharyngeal carcinoma
STIP1: Stress-induced phosphoprotein 1
WGA: Wheat germ agglutinin
ALPPL-2: Alkaline phosphatase placental-like 2
HDLBP: High density lipoprotein binding protein
PI3K: Phosphoinositide 3-kinase
mTOR: Mammalian target of rapamycin
EGFR: Epidermal growth factor receptor
CKM: Creatine kinase, isoform M
MDK: Midkine
FN1: Fibronectin 1
PD: Parkinson’s disease
CSF: Central nervous system
CNS: Central nervous system
PTB: Pulmonary tuberculosis
SAA: Serum amyloid protein A
NPS-PLA2: Secreted phospholipase A2
CA6: Carbonic anhydrase 6
RA: Rheumatoid arthritis
Ems: Extra-articular manifestations
AKI: Acute kidney injury
DMD: Duchenne muscular dystrophy
ERBB4: Erb-B2 receptor tyrosine kinase 4
IPF: Idiopathic pulmonary fibrosis
FVC: Forced vital capacity
DLco: Diffusion capacity of the lung for carbon monoxide
CPI: Composite physiologic index
